# The design of a microprocessor system for automatic signal averaging and percentage purity calculations coupled to a nuclear magnetic resonance spectrometer

**DOI:** 10.1155/S1463924679000262

**Published:** 1979

**Authors:** F. Morley, I. K. O'Neill, M. A. Pringuer, P. B. Stockwell

**Affiliations:** 1 Laboratory of the Government Chemist Cornwall House Stamford Street London SEI 9NQ UK; 2 RTZ Services Ltd. York House Bond Street Bristol BS1 3PE UK; 3 Life Science Research Essex Stock UK


accepts signals from the unknown sample. There are facilities for inserting the standard value and the system automatically carries out the calculation $\frac{\text{test reading}}{\text{std. reading}}$ x standard value. There are also logarithmic facilities available and other linearising facilities have been considered but have not so far been needed. Work is in hand to replace this system and the control unit by a microprocessor.

The entire fluid handling system together with the photometric unit was housed in a steel cabinet well insulated with polystyrene foam and thermostatted at 35° ± 0.2°. The system has been tested by estimating serum glucose with glucose oxidase in conjunction with a chemiluminescent reaction and total serum protein by a biuret method, (Weischelbaum [11]) but operating at 30°.

Chemiluminescence is a phenomenon that deserves more exploitation as a means of making kinetic observations, since the light level obtained is directly proportional to the rate of the reaction. In the method used, liberated hydrogen peroxide was allowed to react with an acridinium salt and the light emitted measured at 425 nm. This method was developed by one of us (DET) and will be fully described elsewhere.

The reproducibility of the glucose method was excellent; 20 replicate aqueous samples of nominally 11.1 mmol/l gave a mean of 11.09 mmol/l and a coefficient of variation of 0.62%. This method is linear to within 1% up to 33.3 mmol/.

46 samples of plasma preserved with fluoride were measured, both by the chemiluminescent and hexokinase methods and gave a correlation coefficient of 0.996. The only disadvantage of the method is the relatively large volume (60 µl) of plasma needed for each test. For measurement of total protein, the machine was used in the absorbance mode with the arithmetic unit equipped with an analogue logarithmic converter. Assays of 20 replicates of bovine albumin 80 g/l gave a mean of 80.33 and a coefficient of variation of 0.45%. It is interesting to note that the error in this analysis is very little more than that found in the sampler itself so that other measuring units make little contribution to the total error. The machine showed no departure from linearity up to 120 g/l. 24 plasma samples with total protein ranging from 42 to 88 g/l were assayed both by the emergency machine and the Vickers M300 and a coefficient of correlation of 0.980 was obtained.

A number of other analytical methods have been tried under simulated conditions but have not yet been used on the machine. These include a fluorimetric rate method for glucose; a fluorimetric rate method for alkaline phosphatase and a urease urea method using conductivity to measure the rate of liberation of ammonia.

ACKNOWLEDGEMENTS
The authors are grateful to Mr. A. Hoare of A. W. Dixon & Co., Avenue Road, Beckenham, Kent, who made all the glassware gratis. Funds for this development were provided by the Wellcome Foundation.

REFERENCES

[1] Pardue, H. L. in *Recent Advances in Analytical Biochemistry* Vol 7, 1969 Interscience.
[2] Malmstadt, H. V., Delaney, C. J., Cordes, E. A. *C.R.C. Critical Reviews in Analytical Chemistry* 1972, **2**, 559.
[3] Moss, D. W. *Clinical Chemistry* 1972, **8**, 1149.
[4] Cook, J. G. H. *Clinica Chimica Acta* 1971, **32**, 485.
[5] Fabiny, D. L., Ertingshausen, G. *Clinical Chemistry* 1971, **17**, 696.
[6] Hewitt, T. E., Pardue, H. L. *Clinical Chemistry* 1973, **19**, 1128.
[7] Kadish, A. H., Little, R. L., Sternberg, J. C. *Clinical Chemistry* 1968, **14**, 116.
[8] Hallett, C. J., Cook, J. G. H. *Clinica Chimica Acta* 1971, **35**, 33.
[9] Arcq, J. C., Arcq, A. *Clinica Chimica Acta* 1973, **48**, 287.
[10] Shipe, J. R., Savory, J. *Clinical Chemistry* 1972, **18**, 1323.
[11] Weichselbaum, T. E. *American Journal of Clinical Pathology*, Technical Section 1946, **10**, 40.

### The design of a microprocessor system for automatic signal averaging and percentage purity calculations coupled to a nuclear magnetic resonance spectrometer

**F. Morley, I. K. O'Neill*, M. A. Pringuer**, and P. B. Stockwell**
*Laboratory of the Government Chemist, Cornwall House, Stamford Street, London SE1 9NQ.*

NUCLEAR magnetic resonance (NMR) spectroscopy is inherently a quantitative technique and the area of a group of peaks is therefore directly related to the concentration. The practical problem is the reliable integration of the area under the peak. Digital integrators are readily available for gas and liquid chromatographic applications but as yet none has been specifically designed for NMR spectroscopy. In contrast to gas chromatography, where gaussian shaped peaks are the ideal, NMR peaks have a considerably faster slew rate and require different integration criteria to measure the correct area, whilst fine structure provides useful identification data it is the total area within the multiplet that is required for quantitation. An integrator specifically designed to measure the area under NMR peaks with the fine structure of the peak retained has been designed by Bunting[1, 2]. This paper describes the application of a microprocessor system to control both the spectrometer and the integrator to measure the areas under selected peaks, the use of multiscan averaging to improve signal to noise characteristics and to automatically calculate the percentage purity of a sample. A single scan of the NMR spectrum exhibits random noise characteristics which can be improved by repetitive scans to the extent of $N^{1/2}$ where N = number of scans.

Methods have been developed in this laboratory to analyse pharmaceuticals quantitatively by NMR and an evaluation of the microprocessor controlled system described in this paper has been published by Pringuer et al [3]. A JEOL 60 $MH_z$ NMR

* *Presently on secondment to RTZ Services Ltd., York House, Bond Street, Bristol BS1 3PE.*
** *Present address, Life Science Research, Stock, Essex.*

spectrometer is used in this work. An internal standard is added to a solution of the pharmaceutical which has a resonance peak near to, but not overlapping the sample reference peak. The percentage purity of the pharmaceuticals is calculated by the microprocessor using the formula set out below. In practice a single spectrum scan is taken; the appropriate data entered into the microprocessor control and once initiated the computer central control handles the repetitive scanning and calculations required.

### General considerations

The percentage purity of each constituent part of the spectrum is calculated by using the following formula:

Percentage purity is equal to

$$\frac{\text{the weight of standard}}{\text{PE weight of standard}} \times \frac{\text{area X}}{\text{area standard}} \times \frac{\text{PE weight of X} \times 100}{\text{weight of X}}$$

Where proton equivalent (PE) weight equals:

$$\frac{\text{molecular weight of sample}}{\text{number of protons in sample peak}}$$

To obtain the percentage purity the values of area X and area standard are measured from the spectrum, and weighings either supplied by the analyst or entered automatically from an electronic balance linked to the control system. In order that the areas are measured accurately they must be precisely defined as to the start and end of the peaks, this is achieved by using the zone controller function on the integrator which allows the analysts to define the locations of up to six peaks or groups of peaks exactly. The measurement of the peak area is performed by measuring the voltage level of the spectrum signal, converting the voltage to a series of pulses using a voltage to frequency convertor and counting the pulses for the duration of the peak. The ratio of peak areas can be calculated using the total pulse count for each peak. To obtain an average peak area for a number of scans two approaches are possible:

(1) to store each area value and average at the end of the run, or
(2) to retain a running average of the peak area which is updated after each successive scan.

For this particular application the first approach was considered unacceptable because it requires a large area of computer memory for data storage, typically six hundred words for 100 scans. The second approach, which requires only six words of storage for any number of scans, was chosen. As memory prices fall this constraint becomes less significant. Calculations must be performed whilst the controller is moving between two successive zones but this processing only requires a few milliseconds and this is easily accomplished.

The running average for each zone is calculated using the following formula:

$$A_N = \frac{A_{(N-1)} \times (N-1) + S_N}{N}$$

N = the number of current scan
$S_N$ = the value of the current sample
$A_{(N-1)}$ = the last average
$A_N$ = the current average.

The precision of all numeric calculations must be within 0.1% and hence 16 bit computation is used since this reduces the number of program steps for any calculation.

The spread of values for the operator supplied parameters ranges from between 9.99 to 999.00. These values are entered prior to the start of an analytical run using a series of thumbwheel switches on the instrument front panel. A five digit fixed point number is entered and automatically rescaled to four digits which avoids the use of floating point arithmetic at this stage.

### Electronic hardware design

The hardware can be discussed as two separate modules,
(a) the digital integrator and
(b) the microprocessor control system.

### The digital integrator

The design of this integrator has been described by Bunting [1,2] and is specifically tailored to NMR applications. It contains a signal amplifier which amplifies the spectrometer signal to 10 volts fullscale. A variable offset system is available which allows the voltage to frequency converter to be zeroed at any different baseline setting. Analog to digital conversion is performed by a

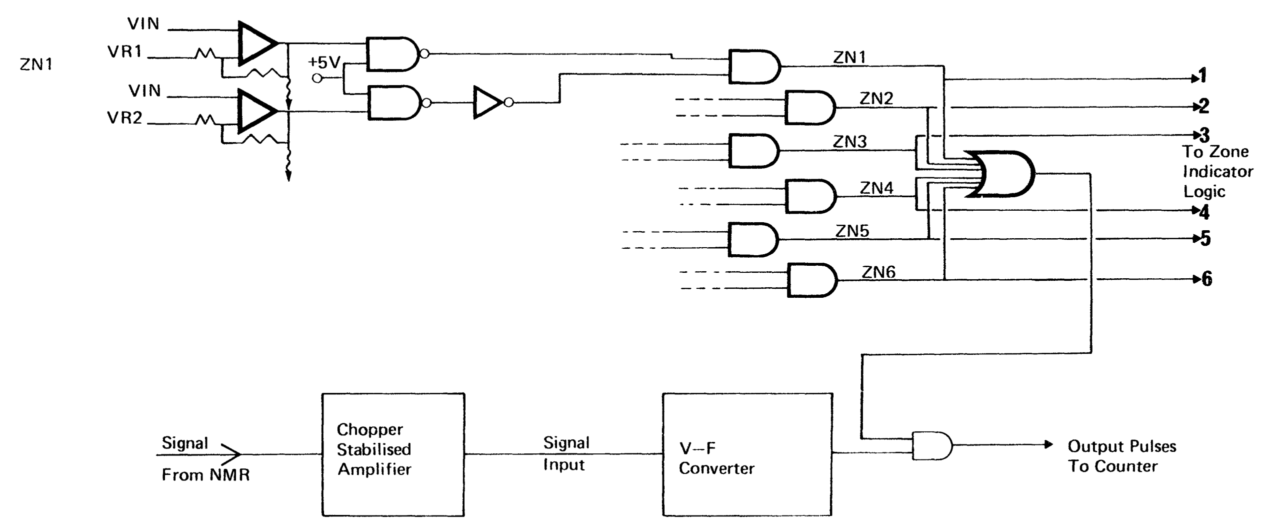

Digital Integrator

ZN 1 – 6 are the zones selected for the NMR Scan.
VR1 & VR2 are the reference voltages derived from the front panel zone setting potentiometers.
V1N is the signal voltage from the linear transducer on the chart recorder.

*Figure 1*

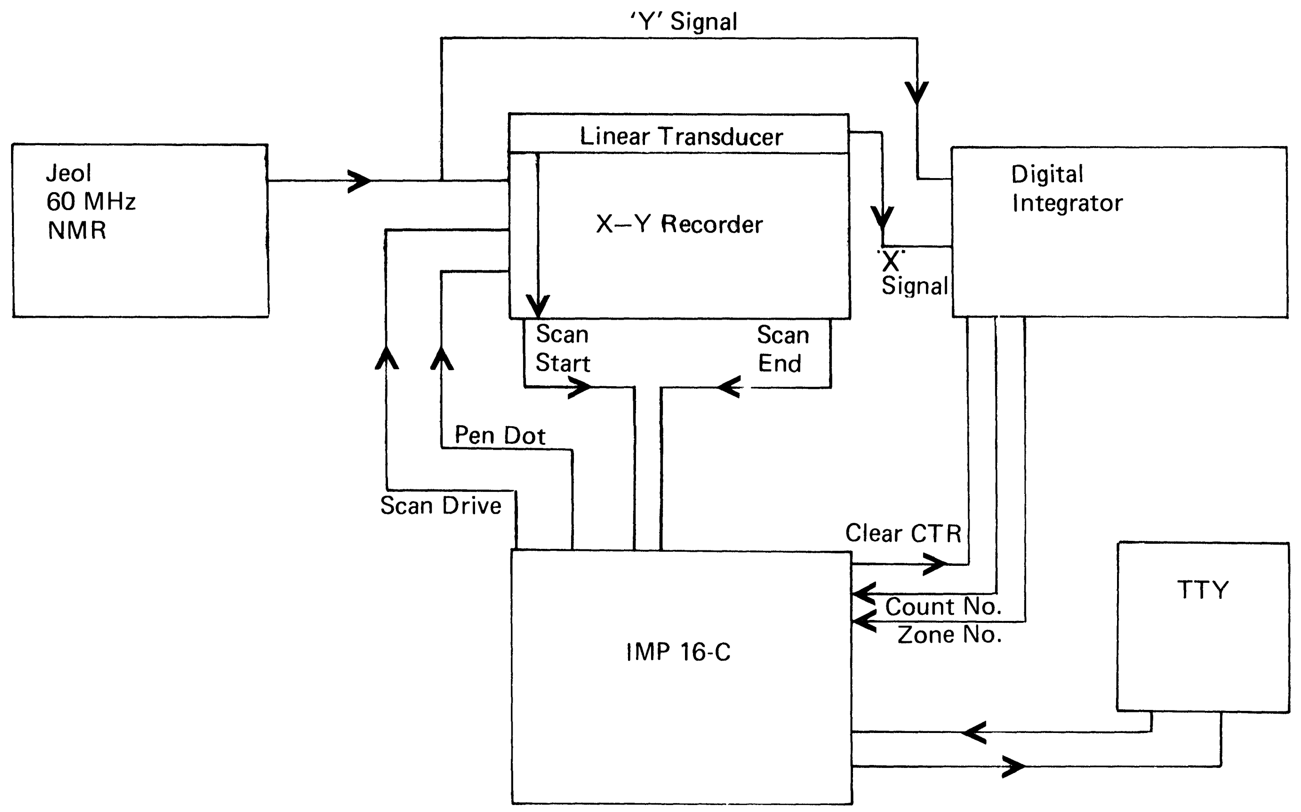

*Figure 2*

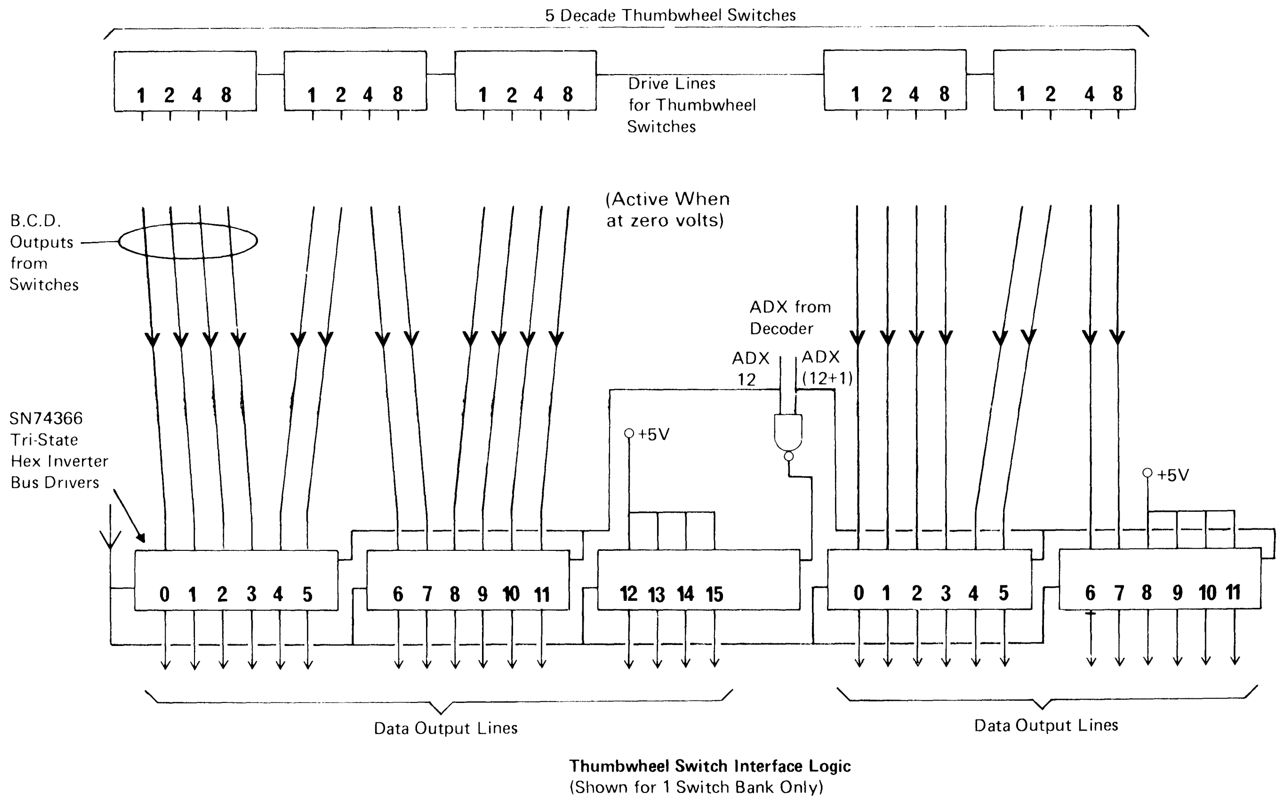

*Figure 3*

voltage to frequency converter and the output pulses are gated into a five decade counter. Counting is only initiated and recorded whilst the NMR scan is within one of the defined zones in the spectrum. This zone definition is obtained by comparing a pre-settable reference voltage with the output voltage from a linear transducer mounted on the chart recorder and linked to the pen carriage. Figure 1 shows the basic components of the integrator. The start and end of each zone is preset by the operator using controls mounted on the front panel, each control has a digital readout which corresponds with the markings on the chart recorder paper. The control system setting corresponds to a resolution of about 1 mm of recorder pen movement on the chart paper.

### The microprocessor control system

The heart of the system is a National Semiconductor IMP 16c-300 central processor unit. This is a 16 bit machine which can address up to 64K words of memory and has an instruction set of some 60 instructions including double length precision arithmetic, 32 bit precision arithmetic can therefore be performed. The operators control panel contains data entry switches, data display LED's and load address, load data, display, execute, clear system and halt switches. The control panel is used for entering the required analytical parameters i.e. weighing and zone parameters and the system start address. Whilst the spectrometer is under microprocessor control the control panel itself is disabled to prevent any unintentional change in parameters or any interference.

The various data and control signals between the microprocessor and the spectrometer and integrator are shown schematically in Figure 2. All other information required for the calculations is entered using the thumbwheel switches mounted on the front panel of the microprocessor system; these include entering the number of scans required, the equivalent weight of the standard zone and the proton weights of all six zones.

The thumbwheel switches are interfaced to the microprocessor using tri-state logic bus drivers as shown in Figure 3.

The zone number is entered as a BCD coded digit from 1-6 which uniquely defines each zone being scanned, the decoding of the zone number outputs into BCD is shown in Figure 4. The count pulses from the voltage frequency converter are fed into a 16 bit binary counter mounted inside the microprocessor system, this provides a binary count equivalent to the decimal count displayed on the integrator, Figure 5. Once initiated the chart recorder is scanned and reset across the spectral range by a command signal from the microprocessor. As the spectrum is recorded under microprocessor control the pen is lifted except to provide the dotting facility to mark the beginning and end of each zone. This facility enables the operator to detect frequency drift in the spectrometer. The scan start and scan end signals are obtained from limit switches on the chart recorder. Figure 2 shows an overall block-schematic of the system.

### Software considerations

The program is written as a turn key system and requires no operator intervention once the initial parameters have been set and the spectral accumulation initiated. The basic flow chart, Figure 6 shows that the average areas for each zone for the total number of scans is calculated on a continuous running average basis. When the total number of scans has been completed, the pre-set parameters are recalled from memory and the percentage purity calculated and printed out on a teletype or similar device. Any software changes that are required in the light of operating experience are made by the design engineer. The program as written occupies approximately IK of memory.

### Results

The system was compared to the normal capacitive integrator providing the standard on the spectrometer. From the results shown in Table 1 it can be shown that the performance of the digital integrator is superior to the capacitive system. The integral ratios and the relative standard deviations for the system developed are both significantly more reproducible than the conventional capacitance integrator. A digital system is not subject to the same leakage and environmental problems associated with the capacitive integrators.

The integration performed is different for each type of integrator but both require some compromise with the ideal operation of the NMR technique. The capacitive integrator averages out any ringing on the peak multiplet and as such the

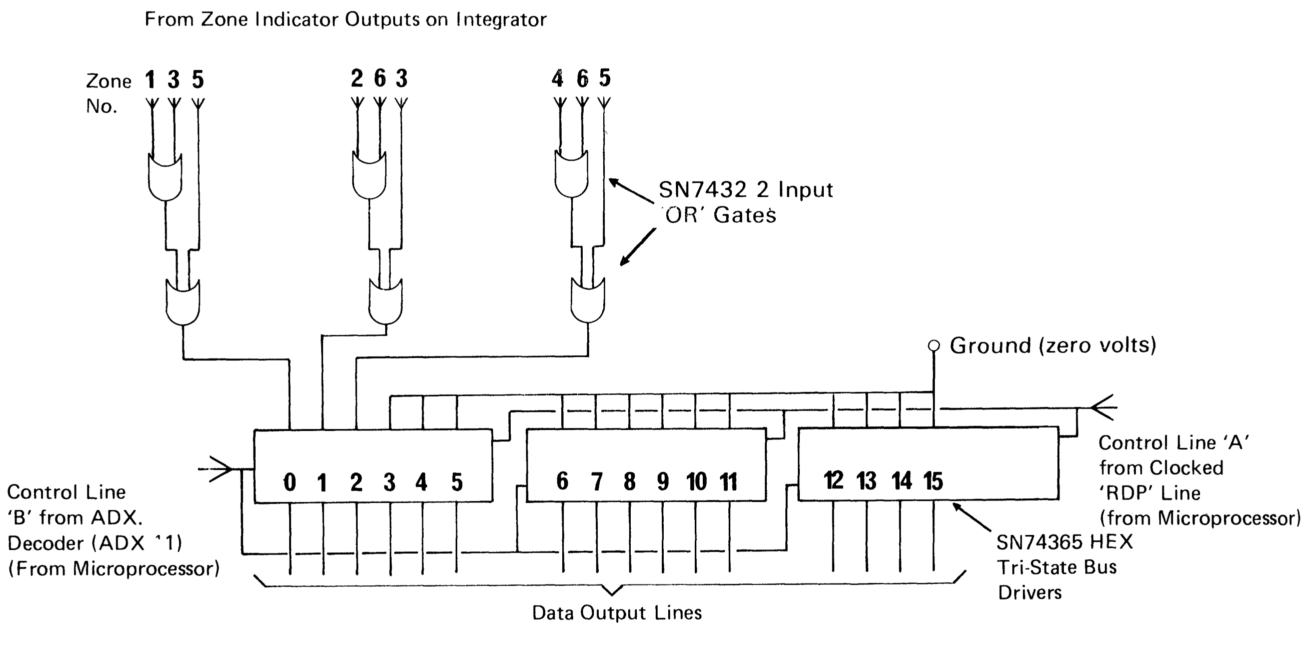

Zone Indicator Logic

SN74365 & SN7432
From Texas Instruments

*Figure 4*

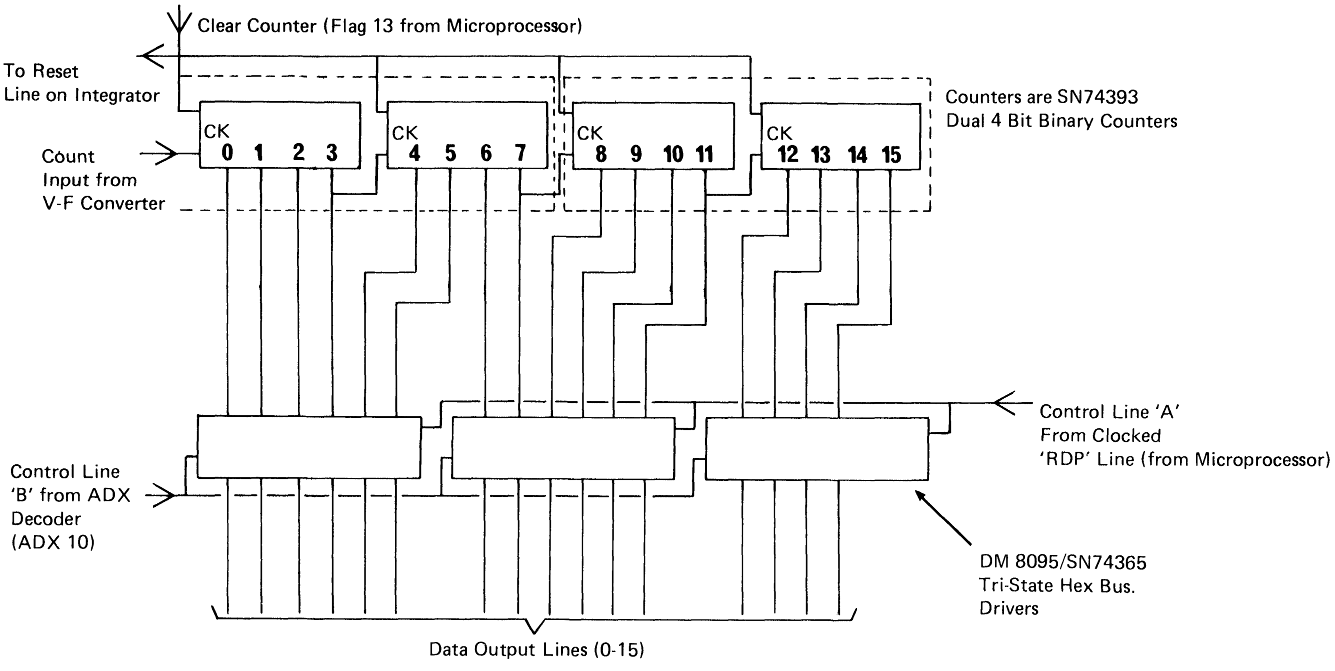

*Figure 5* Binary Area Counter & Tri-State Driver

**Table I Comparison of digital and capacitive integration of ethyl benzene (10 scans)**

| | $C_6H_5$ | $CH_2$ | $CH_3$ |
|---|---|---|---|
| Capacitive | 5 | 1.84 | 2.82 |
| | s = 2.02% | | |
| Digital | 5 | 1.92 | 2.97 |
| | s = 0.57% | | |
| Theoretical | 5 | 2 | 3 |

negative areas partly offset the positive areas in this technique. In contrast the digital integrator will measure only positive areas above the preset baseline. Errors can easily be associated with this approach but these can readily be removed either by adjusting the spectrometer signal to remove any ringing or by precise setting of the zone limits to exclude any significant effects of this ringing feature.

In use the microprocessor system compares favourably with the original system supplied with the spectrometer, but in addition it relieves the operator from time consuming and tedious chart measurements and is also fully automated once the program has been entered and control by the microprocessor initiated.

### Conclusions

The system described has been successfully for two years on the JEOL spectrometer. The addition of the microprocessor system has extended the usefulness of the instrument when its original specification has been out moded by current instrument advances.

REFERENCES

[1] Bunting, W., MSc Thesis, University of London, 1974.

[2] Bunting, W., UK Patent No 1483552, August 1977.

[3] Pringuer, M. A. Morley, F., O'Neill, I. K. and Stockwell, P. B., *Analytical Chemistry* 1978 *in press.*

